# Nocturnal hypoxemic burden and micro- and macrovascular disease in patients with type 2 diabetes

**DOI:** 10.1186/s12933-024-02289-w

**Published:** 2024-06-06

**Authors:** Sarah Driendl, Stefan Stadler, Michael Arzt, Florian Zeman, Iris M. Heid, Mathias Baumert

**Affiliations:** 1 Department of Internal Medicine II, University Hospital Regensburg, Franz-Josef-Strauss-Allee 11, 93053 Regensburg, Germany; 2 Centre of Clinical Studies, University Hospital Regensburg, Franz-Josef-Strauss-Allee 11, 93053 Regensburg, Germany; 3 Department of Genetic Epidemiology, University Hospital Regensburg, Franz-Josef-Strauss-Allee 11, 93053 Regensburg, Germany; 4 Discipline of Biomedical Engineering, School of Electrical and Mechanical Engineering, University of Adelaide, North Terrace, Adelaide, SA 5000 Australia

**Keywords:** Hypoxia, Hypoxemic burden, Cardiovascular disease, Chronic kidney disease, Type 2 diabetes

## Abstract

**Background:**

Micro- and macrovascular diseases are common in patients with type 2 diabetes mellitus (T2D) and may be partly caused by nocturnal hypoxemia. The study aimed to characterize the composition of nocturnal hypoxemic burden and to assess its association with micro- and macrovascular disease in patients with T2D.

**Methods:**

This cross-sectional analysis includes overnight oximetry from 1247 patients with T2D enrolled in the DIACORE (DIAbetes COhoRtE) study. Night-time spent below a peripheral oxygen saturation of 90% (T90) as well as T90 associated with non-specific drifts in oxygen saturation (T90_non − specific_), T90 associated with acute oxygen desaturation (T90_desaturation_) and desaturation depths were assessed. Binary logistic regression analyses adjusted for known risk factors (age, sex, smoking status, waist-hip ratio, duration of T2D, HbA1c, pulse pressure, low-density lipoprotein, use of statins, and use of renin-angiotensin-aldosterone system inhibitors) were used to assess the associations of such parameters of hypoxemic burden with chronic kidney disease (CKD) as a manifestation of microvascular disease and a composite of cardiovascular diseases (CVD) reflecting macrovascular disease.

**Results:**

Patients with long T90 were significantly more often affected by CKD and CVD than patients with a lower hypoxemic burden (CKD 38% vs. 28%, *p* < 0.001; CVD 30% vs. 21%, *p* < 0.001). Continuous T90_desaturation_ and desaturation depth were associated with CKD (adjusted OR 1.01 per unit, 95% CI [1.00; 1.01], *p* = 0.008 and OR 1.30, 95% CI [1.06; 1.61], *p* = 0.013, respectively) independently of other known risk factors for CKD. For CVD there was a thresholdeffect, and only severly and very severly increased T90_non−specific_ was associated with CVD ([Q3;Q4] versus [Q1;Q2], adjusted OR 1.51, 95% CI [1.12; 2.05], *p* = 0.008) independently of other known risk factors for CVD.

**Conclusion:**

While hypoxemic burden due to oxygen desaturations and the magnitude of desaturation depth were significantly associated with CKD, only severe hypoxemic burden due to non-specific drifts was associated with CVD. Specific types of hypoxemic burden may be related to micro- and macrovascular disease.

**Supplementary Information:**

The online version contains supplementary material available at 10.1186/s12933-024-02289-w.

## Introduction

Sleep-disordered breathing (SDB) is characterized by repetitive apneas and hypopneas during sleep, leading to intermittent hypoxemia and recurrent arousals from sleep. It is common in patients with diabetes mellitus type 2 (T2D), and both diabetes and SDB are associated with micro- and macrovascular damage [1–4]. Indeed, SDB and T2D are each likely to contribute to the development of the other [5], showing high coincidences [5, 6]. Chronic kidney disease (CKD), characterized by reduced glomerular filtration rate (GFR) and elevated urine albumin excretion, is a consequence of microvascular damage. Importantly, the prevalence of CKD and mortality has globally increased by 29% and 42%, respectively, since 1990 [7]. About 50–60% of patients wih CKD also suffer from SDB [8] and have an increased risk of end-stage renal disease [9]. A recent meta-analysis demonstrated that the co-existence between sleep apnea and advanced CKD doubled overall mortality [10].

SDB has been linked to hypertension [11], increased risk for incident stroke [12] and increased incidence of total cardiovascular disease (CVD) [13] in meta-analyses in the general population. CVD affects approximately one-third of all patients with T2D [2], but data on the association between SDB and CVD in these patients is sparse. CVD is the primary cause of mortality in patients with T2D, accounting for up to 52% of deaths [14]. The economic burden of CVD in these patients substantially impacts direct medical costs at both the patient and population levels [15].

The above named pathophysiological mechanisms of SDB are central in CVD processes through activation of various intermediate pathways including sympathetic activation, inflammation, oxidative stress, metabolic dysregulation, and mechanical stress [16–19]. SDB is diagnosed by an elevated apnea-hypopnea index (AHI, number of hypopneas and apneas per hour of sleep). But it encompasses a broad variation in nocturnal hypoxemia [20, 21] that may not be reflected in AHI, as this parameter does not distinguish between brief or sustained changes in peripheral oxygen saturation (SpO_2_) or the depth of desaturations or the baseline SpO_2_ level from which they occur. These cumulative effects may be clinically important [22] as recent studies suggest that nocturnal hypoxemic burden is an independent predictor of cardiovascular mortality [23] and all-cause mortality in stable chronic heart failure [24] and is more strongly associated with adverse cardiovascular outcomes than the AHI [25]. Also, factors other than SDB may contribute to nocturnal hypoxemic burden, such as COPD or heart failure, and contribute to micro- and macrovascular damage [26].

The present study aimed to characterize the composition of nocturnal hypoxemic burden in patients with T2D and to assess its association with micro- and macrovascular disease. We hypothesized that specific oximetry-derived parameters of nocturnal hypoxemic burden are associated with chronic kidney disease and cardiovascular disease in patients with T2D.

## Patients and methods

### Study population

We investigated participants of the DIACORE (DIAbetes COhoRtE)-SDB (sleep-disordered breathing) sub-study [27]. DIACORE is a prospective, longitudinal, two-center cohort study of 3000 T2D patients of European descent in the cities and counties of Regensburg and Speyer [28]. DIACORE participants of the Regensburg study center who did not use a positive airway pressure device were invited to participate in the DIACORE-SDB sub-study. Patients with chronic obstructive pulmonary disease (COPD) were not excluded. A total of 1491 patients agreed to participate in the sub-study; the baseline survey was conducted between 2010 and 2014 [27]. The protocol, the data protection strategy, and the study procedures were approved by the Ethics Committees of the participating institutions and were in accordance with the Declaration of Helsinki.

### Quantification and characterization of nocturnal hypoxemic burden

Hypoxemic burden was assessed using a validated 2-channel ambulatory monitoring device [29] recording nasal flow and pulse oximetry (ApneaLink®, ResMed) as described previously [27]. Participants were instructed how to use the device by trained personnel in a standardized manner [27]. Oximetry signals were extracted for further processing using a fully automated and custom-made computer algorithm programmed in MATLAB® (MathWorks®, Natick, MA, USA), as described previously [30]. Signal artifacts were automatically detected and excluded based on a set of empirical criteria (e.g. instantaneous changes in SpO_2_ > 5%) [30].

Nocturnal hypoxemic burden was defined as analyzed recording time spent at SpO_2_ levels below 90% (T90) in minutes. To further characterize the composition of nocturnal hypoxemic burden, we quantified the component of T90 associated with non-specific and non-cyclic drifts in SpO_2_ or incomplete resaturation (T90_non−specific_) versus T90 associated with acute oxygen desaturation events accompanied by resaturation (T90_desaturation_) [23, 30]. Acute desaturations were defined as episodic, monotonic drops in oxygen saturation by at least 4% from any prior level that was followed by a resaturation to at least two-thirds of the oxygen saturation level observed before desaturation within 150 s starting from the onset of desaturation [23]. Oxygen desaturation index (ODI) measured the mean number of respiratory events per hour where blood oxygen level dropped by 4% compared to immediately preceding basal value. We measured the desaturation depth as the average SpO_2_ reduction throughout acute events (in %) and obtained the median value across all desaturation events. Apnea-hypopnea index (AHI) was calculated as the mean number of apnea and hypopnea events per hour of recording time.

### Assessment of macro—and microvascular disease

The participants completed a standardized online questionnaire and underwent physical examination [28]. Arterial blood pressure was determined via repeated measurement: three measures were performed, and the last two were averaged [28]. Pulse pressure was calculated as the difference between systolic and diastolic blood pressure. Whole blood samples were drawn after the patients had rested in a seated position for at least 15 min [28]. Estimated GFR was calculated using the CKD-EPI equation from 2009 [28]. Microvascular disease was assessed via serum creatinine, GFR, and urine albumin-creatinine ratio (uACR) as surrogates for renal function. CKD was defined as elevated urine albumin excretion (uACR > 30 mg/g), reduced eGFR (< 60 ml/min per 1.73 m^2^), or both, following current KDIGO guidelines [31]. CVD was determined as a composite of peripheral artery disease (PAD), coronary artery disease (CAD), or stroke. PAD and CAD were ascertained by self-reported history of surgical or interventional therapy of PAD or CAD or myocardial infarction and validated from medical records and direct contact with local physicians [32]. Stroke was defined as a self-reported history of at least one ischemic or embolic stroke ascertained by a review of a physician’s record, including cerebral imaging. The stroke definition did not include the diagnosis of carotid stenosis, transient ischemic attacks, or intracerebral bleeding [32]. The wording of the questions asked in the questionnaire can be found in the online supplement (S1).

### Statistical analysis

Descriptive data are presented as mean (± standard deviation) for normally distributed variables and median [interquartile range] otherwise. Group comparisons of continuous variables were performed by t-test for normally distributed data, Mann-Whitney-U-test for non-normally distributed variables, and Chi-square test for categorical variables. We used logistic regression to analyze the association between hypoxemic burden and CKD and CVD. Known confounders for atherosclerosis and chronic renal disease, such as sex, age, waist-hip ratio (WHR), pulse pressure, duration of T2D, HbA1c, low-density lipoprotein (LDL), smoking status (current or former vs. never smokers), use of statins, and use of renin-angiotensin-aldosterone system inhibitors were included as covariates. Results are presented as odds ratio (OR) estimates with a 95% confidence interval (CI). We performed a Bonferroni correction for two independent tests and a stricter level of significance was considered statistically significant, i.e. P values < 0.05/2 = 0.025. Data were analyzed using the SPSS statistical software package (SPSS 28.0 IBM SPSS Statistics, Armonk, New York, USA).

## Results

### Patient characteristics

**Fig. 1:**
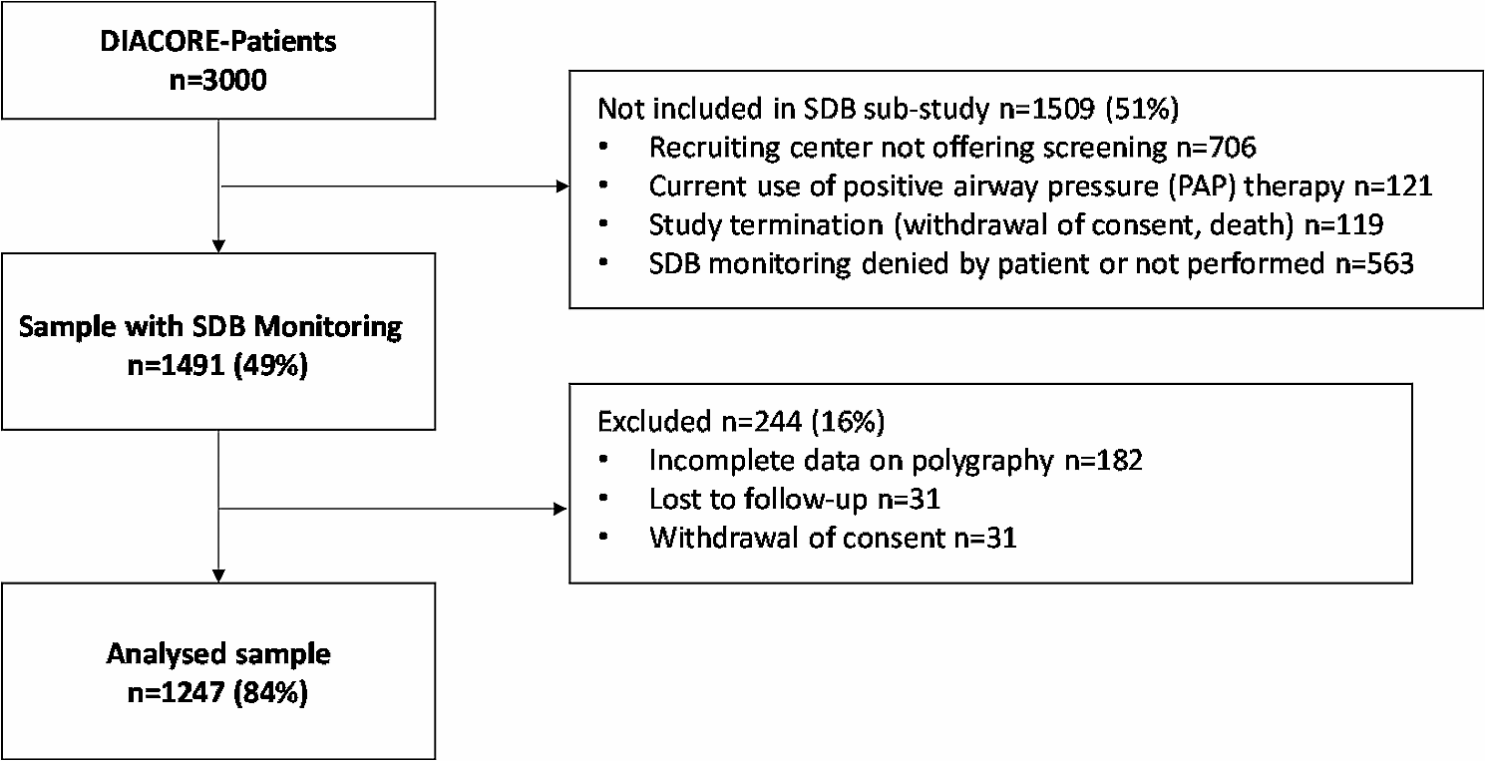
Study flow chart

Complete data were available for 1247 patients (83.6%) of the 1491 DIACORE-SDB sub-study participants. 244 (16.4%) patients were excluded from the final analysis due to incomplete polygraphy data, loss to follow-up or withdrawal of consent (Fig. 1). Characteristics of the analyzed cohort are summarized in Table 1. The median T90, T90_desaturation_, and T90_non−specific_ were 16.0 [2.1; 57.4] min, 3.5 [0.2; 34.1] min and 6.5 [1.1; 21.1] min, respectively. The median ODI was 6.6/h [2.7; 14.9] and the median desaturation depth was 2.7% [2.5; 3.1].

**Table 1:** Baseline characteristics of the 1247 patients, overall and according to the severity of the hypoxemic burden

Variables	Entire cohort	T90 Q1-2 [0–16.0 min]	T90 Q3–4 [> 16.0 min]	*P* value	T90_non−specific_ Q1-2 [0–3.5 min]	T90_non−specific_ Q3-4 [> 3.5 min]	*P* value	T90_desaturation_ Q1-2 [0–6.5 min]	T90_desaturation_ Q3-4 [> 6.5 min]	*P* value
n (%)	1247	623 (50)	624 (50)		622 (50)	625 (50)		622 (50)	625 (50)	
Age [years]	67 ± 9	65 ± 9	68 ± 8	< 0.001	65 ± 9	68 ± 8	< 0.001	65 ± 9	68 ± 8	< 0.001
Sex (female), n (%)	514 (41.2)	288 (46.2)	226 (36.2)	< 0.001	275 (44.2)	239 (38.2)	0.032	284 (45.7)	230 (36.8)	0.001
BMI [kg/m^2^]	30.9 ± 5.4	29.6 ± 5.1	32.2 ± 5.4	< 0.001	30.0 ± 5.2	31.9 ± 5.4	< 0.001	29.7 ± 5.1	32.2 ± 5.4	< 0.001
Waist-hip ratio	0.95 ± 0.08	0.93 ± 0.08	0.97 ± 0.08	< 0.001	0.94 ± 0.08	0.97 ± 0.08	< 0.001	0.94 ± 0.08	0.97 ± 0.08	< 0.001
Former or current smokers, n (%)	704 (56.5)	323 (51.8)	381 (61.1)	< 0.001	337 (54.2)	367 (58.7)	0.106	319 (51.3)	385 (61.6)	< 0.001
High alcohol intake, n (%)	358 (28.7)	171 (47.8)	187 (52.2)	0.325	164 (26.4)	194 (31.0)	0.068	155 (24.9)	203 (32.5)	0.003
Physical inactivity, n (%)	701 (56.2)	317 (45.2)	384 (54.8)	< 0.001	319 (51.3)	382 (61.1)	< 0.001	321 (51.6)	380 (60.8)	0.001
HbA1c [mmol/mol]	54 ± 16	48 [43; 54]	50 [43; 57]	0.020	48 [43; 54]	49 [43; 56]	0.070	48 [43; 54]	50 [44; 56]	0.010
T2D duration [years]	8.0 [3.9; 14.0]	7.5 [3.4; 13.7]	8.7 [4.3; 14.9]	0.015	7.7 [3.6; 13.8]	8.2 [4.2; 14.7]	0.125	7.3 [3.6; 13.2]	8.9 [4.2; 15.2]	0.003
Systolic BP [mmHg]	138 ± 18	137 ± 18	140 ± 18	0.002	138 ± 18	140 ± 18	0.048	137 ± 17	140 ± 18	0.001
Diastolic BP [mmHg]	75 ± 10	76 ± 10	76 ± 10	0.493	76 ± 10	76 ± 10	0.673	75 ± 10	76 ± 10	0.381
Pulse pressure [mmHg]	63 ± 15	61 ± 15	64 ± 15	0.002	62 ± 16	64 ± 15	0.011	62 ± 15	64 ± 16	0.001
RAAS inhibition use	813 (65.2)	372 (59.7)	441 (70.1)	< 0.001	369 (59.3)	444 (71.0)	< 0.001	379 (60.9)	434 (69.4)	0.002
Hypertension, n (%)	545 (43.7)	250 (40.1)	295 (47.4)	0.009	261 (42.0)	284 (45.6)	0.197	247 (39.8)	298 (47.8)	0.005
Serum creatinine [mg/dl]	0.88 [0.75; 1.05]	0.85 [0.73; 1.02]	0.90 [0.78; 1.08]	< 0.001	0.87 [0.73; 1.02]	0.89 [0.78; 1.07]	0.003	0.86 [0.73; 1.02]	0.90 [0.78; 1.08]	< 0.001
eGFR-CDKepi [ml/min/1,73 m²]	83 [67; 93]	85 [70; 95]	80 [65; 92]	< 0.001	86 [70; 95]	81 [66; 92]	< 0.001	85 [70; 95]	80 [66; 92]	< 0.001
uACR [mg/g]	9.0 [4.5; 26.6]	8.1 [4.2; 20.9]	10.5 [4.8; 36.5]	< 0.001	7.9 [4.0; 19.6]	10.6 [5.0; 35.9]	< 0.001	8.0 [4.2; 19.5]	10.9 [4.8; 35.9]	< 0.001
LDL [mg/dl]	119 ± 37	122 ± 35	116 ± 38	0.005	121 ± 36	116 ± 37	0.004	122 ± 36	117 ± 37	0.023
HDL [mg/dl]	54 ± 16	55 ± 15	52 ± 16	0.003	55 ± 15	53 ± 16	0.046	55 ± 16	53 ± 15	0.075
Statin use, n (%)	560 (44.9)	245 (40.8)	306 (49.0)	0.003	244 (39.2)	316 (50.6)	< 0.001	264 (42.4)	296 (47.4)	0.081
Mean SpO_2_ [%]	92.4 ± 2.0	93.6 ± 1.2	91.3 ± 1.6	< 0.001	94 ± 1	91 ± 2	< 0.001	93 ± 1	91 ± 2	< 0.001
Min SpO_2_ [%]	81 [78; 83]	82 [81; 86]	80 [76; 82]	< 0.001	82 [80; 85]	80 [76; 82]	< 0.001	82 [81; 86]	79 [76; 82]	< 0.001
Sleep efficiency [%]	0.98 [0.88; 1.00]	0.99 [0.88; 1.00]	0.98 [0.88; 1.00]	0.322	0.98 [0.88; 1.00]	0.98 [0.88; 1.00]	0.066	0.98 [0.88; 1.00]	0.98 [0.88; 1.00]	0.857
T90 [min]	16.0 [2.1; 57.4]	2.1 [0.4; 6.5]	57.3 [28.9; 140.8]	< 0.001	2.1 [0.4; 7.6]	55.7 [25.1; 139.3]	< 0.001	2.1 [0.4; 6.5]	49.5 [21.4; 132.8]	< 0.001
T90_non−specific_ [min]	3.5 [0.2; 34.1]	0.2 [0.0; 1.5]	33.7 [10.0 98.6]	< 0.001	0.2 [0.0; 0.8]	33.3 [10.9; 98.1]	< 0.001	0.3 [0.0; 3.6]	19.1 [3.5; 84.0]	< 0.001
T90_desaturation_ [min]	6.5 [1.1; 21.1]	1.4 [0.3; 4.3]	21.1 [11.2; 40.8]	< 0.001	1.5 [0.3; 6.5]	16.8 [6.4; 37.7]	< 0.001	1.1 [0.2; 3.1]	21.1 [12.2; 40.7]	< 0.001
Desaturation depth [%]	2.7 [2.5; 3.1]	2.6 [2.4; 2.8]	2.9 [2.6; 3.4]	< 0.001	2.6 [2.4; 2.9]	2.8 [2.5; 3.2]	< 0.001	2.5 [2.3; 2.7]	3.0 [2.7; 3.4]	< 0.001
ODI [events/h]	9 [5; 18]	7 [4; 12]	15 [8; 26]	< 0.001	8 [4; 16]	11 [6; 21]	< 0.001	3 [1; 6]	14 [8; 22]	< 0.001
AHI [events/h]	10 [5; 18]	6 [3; 10]	15 [7; 25]	< 0.001	7 [4; 14]	11 [6; 21]	< 0.001	5 [3; 9]	16 [10; 26]	< 0.001
Excessive daytime sleepiness, n (%)	89 (7.1)	40 (8.0)	49 (7.9)	0.334	44 (6.2)	45 (8.4)	0.334	40 (6.4)	49 (7.9)	0.326

Results are provided as mean ± standard deviation for normally distributed and as median [interquartile range] for non-normally distributed variables. High alcohol intake defined as ≥ 3/drinks per week; excessive daytime sleepiness defined as Epworth Sleepiness Scale ≥ 11; physical inactivity defined as light activity ≤ 2 times/week; hypertension defined as blood pressure ≥ 140/90 mmHg; sleep efficiency: sleeping time per time in bed

*RAAS* Renin-angiotensin-aldosterone system, * AHI* apnoea-hypopnoea index, *BMI* body-mass index, *BP* blood pressure, *eGFR-CDKepi* estimated glomerular filtration rate calculated using the CKD-EPI equation, *HbA1c* hemoglobin A1c, *HDL* high density lipoprotein, *LDL* low density lipoprotein,* ODI* oxygen-desaturation index, SpO_2_ arterial oxygen saturation, *T90* night-time spent with oxygen saturation < 90%, T90_desaturation_ T90 associated with acute oxygen desaturation events accompanied by resaturation, T90_non−specific_ T90 associated with non-specific and non-cyclic drifts in SpO_2_ or incomplete resaturation, uACR: urine albumin-creatinine ratio, *Q* quartile

Patients with T90 above the median were predominantly male, older, and more often obese than patients with T90 below the median. They were also more often current or former smokers and more often physically inactive. Diabetes duration, HbA1c, and systolic blood pressure were significantly higher in these patients. LDL, however, was significantly lower. When we analyzed patient characteristics separately for hypoxemic burden due to acute oxygen desaturation events accompanied by resaturation (T90_desaturation_), we observed results similar to T90 (Table 1). For hypoxemic burden due to non-specific drifts (T90_non−specific_), there was no difference in diabetes duration, HbA1c, hypertension, and smoking status between groups (Table 1).

As expected, patients with T90 above the median exhibited significantly higher AHI and ODI values. When analyzing these parameters separately for T90_non−specific_ and T90_desaturation_, results were similar (Table 1). For all hypoxemic burden parameters, patients above the median exhibited significantly higher desaturation depth values than patients in the lowest two quartiles. Patients with ODI ≥ 15/h had similar characteristics to the patients with T90 above the median, but there was no difference between LDL, HbA1c, and uACR for patients with ODI </≥ 15/h (Table S2). For patients with desaturation depth above the median there was no difference with regards to age, smoking status, and physical activity compared to desaturation depth below the median (Table S2).

### Association of hypoxemic burden and chronic kidney disease

**Fig. 2:**
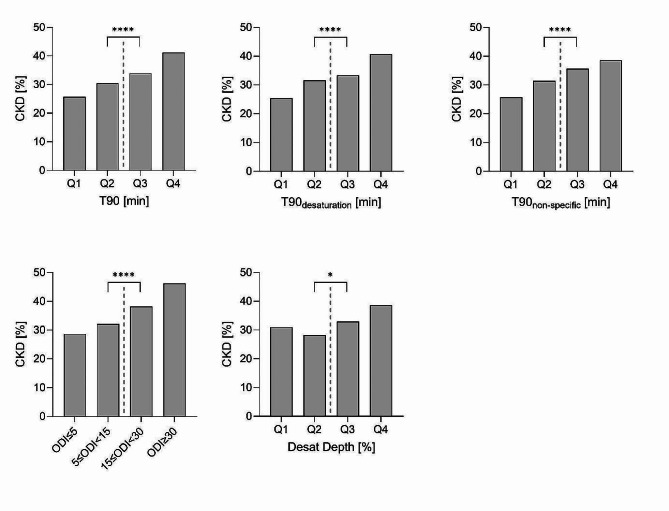
Prevalence of chronic kidney disease according to the presence of hypoxemic burden for T90, T90_non−specific_ and T90_desaturation_, ODI, and desaturation depth. *CKD* chronic kidney disease,* Desat Depth* Desaturation depth, *ODI* oxygen desaturation index,* T90* night-time spent with oxygen saturation < 90%, *T90 desaturation* T90 associated with acute oxygen desaturation events accompanied by resaturation,* T90 non-specific* T90 associated with non-specific and non-cyclic drifts in SpO2 or incomplete resaturation, *Q* quartile. * p<0.05, **** p<0.0001

Patients with T90 above the median had significantly higher serum creatinine levels, lower GFR, and higher uACR (Table 1). In total, 409 (32.8%) patients had CKD; the prevalence was higher in patients with T90 above versus below the median (234 (37.5%) vs. 175 (28.1%), *p* < 0.001). Similar results were obtained when decomposing T90 into T90_non−specific_ and T90_desaturation_. CKD prevalence, serum creatinine levels and uACR were higher and GFR lower in patients above the median for T90_non−specific_ and T90_desaturation_ (Table 1; Fig. 2). The prevalence of CKD also was significantly higher in patients with ODI ≥ 15/h than in patients with ODI < 15/h (40.6% vs. 30.2%, *p* < 0.001) and in patients with desaturation depth above the median versus below the median (35.8% vs. 29.5%, *p* = 0.021).

In univariable logistic regression models, high hypoxemic burden was consistently associated with CKD, considering continuous and dichotomized T90 variables as well as ODI and desaturation depth. To further analyze the elevated odds, we assessed the association between hypoxemic burden and CKD by adjusting the regression models for known risk factors for CKD: age, sex, WHR, smoking status, pulse pressure, duration of T2D, Hb1Ac, use of statins, and use of renin-angiotensin-aldosterone system inhibitors. In the adjusted model, continuous T90_desaturation_ remained significantly associated with CKD but not T90 or T90_non−specific_ (Table 2). We also observed significant associations between hypoxemic burden variables and serum creatinine, GFR, and uACR separately with linear regression models (Table S3). Continuous ODI was associated with CKD in univariable logistic regression, but not in the multivariable model (Table 2). Desaturation depth was significantly associated with CKD independently of the above-named risk factors (Table 2). We adjusted for WHR in the multivariable analyses because it is a better measure of abdominal adiposity. However, when adjusting for body-mass index instead of WHR results were similar (data not shown).

**Table 2:** Multivariate logistic regression models for the association between hypoxemic burden parameters and chronic kidney disease

Variable	Odds ratio (95% CI), multivariate	*P* value
T90 cont.	1.00 (1.00; 1.00)	0.246
T90 Q12 vs. Q34	1.15 (0.89; 1.50)	0.284
T90_non−specific_ cont.	1.00 (1.00; 1.00)	0.688
T90_non−specific_ Q12 vs. Q34	1.15 (0.89; 1.49)	0.295
T90_desaturation_ cont.	1.01 (1.00; 1.01)	**0.008**
T90_desaturation_ Q12 vs. Q34	1.10 (0.85; 1.43)	0.472
Desaturation depth cont.	1.30 (1.06; 1.61)	**0.013**
Desaturation depth Q12 vs. 34	1.18 (0.91; 1.53)	0.202
ODI cont.	1.01 (1.00; 1.02)	0.044
ODI < vs. ≥ 15/h	1.29 (0.97; 1.71)	0.085
ODI < vs. ≥ 30/h	1.40 (0.89; 2.21)	0.144

*ODI* oxygen-desaturation index, *T90* night-time spent with oxygen saturation < 90%, T90_desturation_ T90 associated with acute oxygen desaturation events accompanied by resaturation, T90_non−specific_ T90 associated with non-specific and non-cyclic drifts in SpO_2_ or incomplete resaturation, *Q* quartile

^a^Multivariate analyses adjusted for age, sex, waist-hip ratio, smoking status, HbA1c, diabetes duration, pulse pressure, statin use, and renin-angiotensin-aldosterone system inhibitor use

Bold values statistically significant

**Table 3:** Multivariate logistic regression models for the association between hypoxemic burden parameters and cardiovascular disease

Variable	Odds ratio (95% CI), multivariate^a^	*P* value
T90 cont.	1.00 (1.00; 1.00)	0.439
T90 Q12 vs. Q34	1.20 (0.89; 1.63)	0.238
T90_non−specific_ cont.	1.00 (1.00; 1.00)	0.683
T90_non−specific_ Q12 vs. Q34	1.51 (1.12; 2.05)	**0.008**
T90_desaturation_ cont.	1.00 (1.00; 1.01)	0.356
T90_desaturation_ Q12 vs. Q34	0.86 (0.63; 1.16)	0.315
Desaturation depth cont.	0.91 (0.70; 1.16)	0.438
Desaturation depth Q12 vs. 34	0.90 (0.68; 1.20)	0.479
ODI cont.	1.00 (0.99; 1.01)	0.947
ODI < vs. ≥ 15/h	0.96 (0.68; 1.34)	0.795
ODI < vs. ≥ 30/h	1.20 (0.72; 2.02)	0.480

*HbA1c* hemoglobin A1c, *LDL* low density lipoprotein, T90: night-time spent with oxygen saturation < 90%, T90_desaturation_ T90 associated with acute oxygen desaturation events accompanied by resaturation, T90_non−specific_ T90 associated with non-specific and non-cyclic drifts in SpO_2_or incomplete resaturation, Q quartile

^a^Multivariate analyses adjusted for age, sex, smoking status, waist-hip ratio, HbA1c, diabetes duration, pulse pressure, LDL, statin use, and renin-angiotensin-aldosterone system inhibitor use

Bold values statistically significant

### Association of hypoxemic burden and cardiovascular disease

**Fig. 3:**
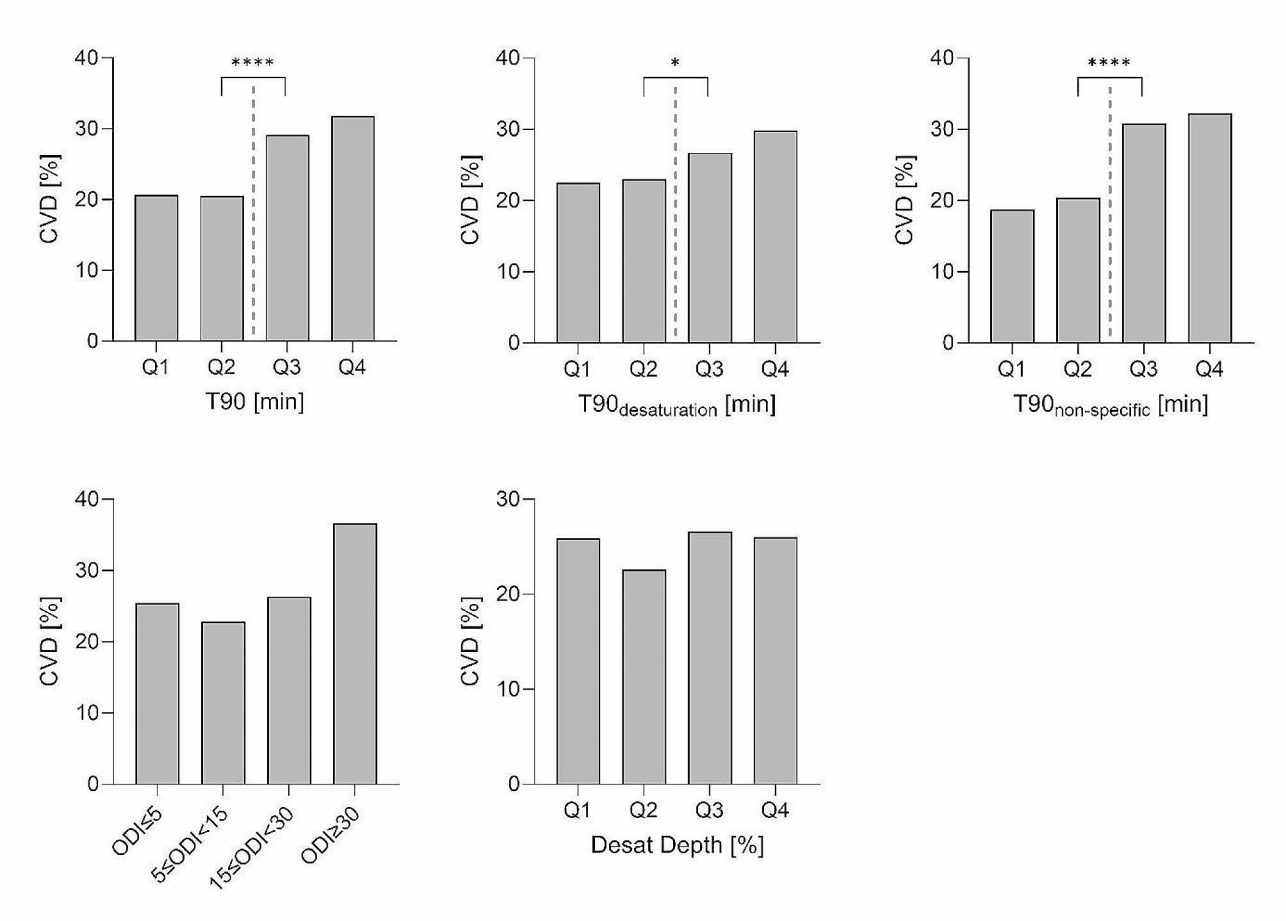
Prevalence of cardiovascular disease according to the presence of hypoxemic burden for T90, T90_non−specific_ and T90_desaturation_, ODI, and desaturation depth. *CVD* cardiovascular disease, *Desat Depth* Desaturation depth,* ODI* oxygen desaturation index, *T90* night-time spent with oxygen saturation <90%, *T90 desaturation* T90 associated with acute oxygen desaturation events accompanied by resaturation, *T90 non-specific* T90 associated with non-specific and non-cyclic drifts in SpO_2_or incomplete resaturation, *Q* quartile. * p<0.05, **** p<0.0001

A total of 318 (25.5%) patients reported a history of CVD, including 244 (19.6%) cases of CAD, 45 (3.6%) cases of PAD, and 77 (6.2%) cases of stroke. CVD was more frequent in patients with T90 above the median (190 (30.4%) vs. 128 (20.5%) patients, *p* < 0.001). Similarly, CVD was more common in patients with T90_non−specific_ above the median (178 (33.1%) vs. 140 (19.7%) patients, *p* < 0.001) and with T90_desaturation_ above the median (211 (28.1%) vs. 107 (21.6%) patients, *p* = 0.011, Fig. 3). There was no significant difference in CVD prevalence between patients with ODI ≥ vs. < 15/h (91 (29.4%) vs. 227 (24.2%), *p* = 0.073) and between patients with desaturation depth above vs. below the median (158 (26.3%) vs. 149 (24.2%), *p* = 0.399).

Univariable logistic regression analysis showed an association between all hypoxemic burden variables and CVD. After adjusting the models for known risk factors for CVD, namely age, sex, WHR, smoking status, duration of T2D, HbA1c, pulse pressure, LDL, use of statins, and use of renin-angiotensin-aldosterone system inhibitors, there was an independent association between dichotomized T90_non−specific_ and CVD.

## Discussion

This cross-sectional analysis of a large cohort of T2D patients showed an association between nocturnal hypoxemic burden and micro- and macrovascular diseases. The prevalence of CKD and CVD was significantly higher in patients with a high nocturnal hypoxemic burden than in those with a lower hypoxemic burden. T90_desaturation_ and desaturation depth were associated with CKD, T90_non−specific_ was associated with CVD. The characteristics of hypoxemic burden may differ between CKD and CVD.

### Association of hypoxemic burden and chronic kidney disease

In our study cohort, nocturnal SpO2 dropped under 90% in 1014 patients (81%) and 310 of the patients had an ODI ≥ 15/h, showing that nocturnal hypoxemic burden is highly prevalent in T2D. The prevalence of CKD in the present study (32.8%) is comparable to other studies including T2D patients of similar age [33, 34].

Different pathophysiological mechanisms by which hypoxemia could exacerbate the risk of developing and worsening CKD have been proposed. Decreased partial oxygen pressure in renal tissue drives renal inflammation, oxidative stress, fibrosis, and subsequent declines in kidney function [35]. Nocturnal hypoxemia contributes to the development of CKD by hypoxia itself and glomerular hypertension/hyperfiltration, increased activation of the sympathetic nervous system and the renin-angiotensin-aldosterone system [36, 37]. In a mouse model, chronic intermittent hypoxia contributed to renal damage and dysfunction by promoting persistent renal hypoperfusion and tissue hypoxia during wakefulness and sleep [38]. GFR was decreased in a study of rats exposed to long-term chronic intermittent hypoxia [39].

In the present study, the hypoxemic burden due to acute, sleep apnea related desaturations expressed by T90_desaturation_ and desaturation depth were associated with CKD independently of known risk factors for CKD. Previous studies observed an association between SDB and CKD. A meta-analysis of seven studies of patients with T2D demonstrated an association between obstructive sleep apnea (OSA), assessed using either AHI or ODI and diabetic kidney disease (pooled OR 1.59, 95% CI [1.16; 2.18]) [40]. Another meta-analysis of six clinical trials and observational studies comprising 29% T2D patients also found an association between sleep apnea and CKD (pooled adjusted OR 2.088 [1.78–2.45] [41].

Considering T90, Marrone et al. found that GFR ≥ 60 ml/min was less prevalent in patients with high hypoxemic burden (T90 > 12%) than in patients with GFR < 60 ml/min [42]. A recent study of 1,295 adults with suspected SDB (comprising 18.5% of patients with T2D) observed an increased risk of CKD progression in patients with severe OSA compared to those with no or mild OSA (OR 2.96 [2.04; 4.30]) [43]. Neither eGFR nor uACR was significantly associated with T90.

In a study by Sakaguchi et al., T90 was an independent predictor of a rapid decline in kidney function in 120 patients [44]. Both severe and moderate hypoxemia were associated with greater glomerular pressure, a sign of increased renal risk [36] and severe hypoxemia was associated with greater renal renin-angiotensin-aldosterone system activity [37]. The question arises as to why, in the present study, only T90_desaturation_ and not T90 was associated with CKD. Studies suggest that as a response to continuous hypoxia, coordinated upregulation of HIF-1 and HIF-2 mediates adaptive responses in the systemic circulation, whereas chronic intermittent hypoxia triggers an imbalance between HIF-1 and HIF-2 activity that leads to oxidative stress, resulting in maladaptive responses [45]. Furthermore, hypertension is one of the main reasons for CKD [31], and SDB can cause hypertension [11]. While the overall T90 was not independently associated with CKD in our study, the component related to acute desaturation events was, suggesting that SDB is the primary driver affecting kidney function rather than other causes of hypoxemic burden, such as obesity and COPD. Supporting this view, the desaturation depth was independently associated with CKD.

As SBD likely contributes to CKD, continuous positive airway pressure (CPAP) therapy may reduce its impact. In a non-randomized study, CPAP treatment significantly reduced the rate of eGFR decline after 12 months, especially in patients with moderate and severe OSA [46]. However, in another randomized controlled trial involving 57 patients, CPAP therapy did not significantly slow the decline in eGFR or reduce albuminuria over 12 months [47]. Yet some improvement in eGFR occurred with CPAP therapy in patients with a lower risk of CKD progression, but this did not reach statistical significance [47]. Larger longitudinal trials investigating the effect of CPAP on CKD are warranted.

### Association of hypoxemic burden and cardiovascular disease

In the present study, 25.5% of the patients reported a history of CVD comparable to other studies on patients with T2D [1, 2]. In the present study, T90 due to non-specific drifts was significantly and independently associated with prevalent CVD. Contrary to CKD, where we observed a dose-response relationship (Fig. 2), for CVD there was a thresholdeffect and only severly and very severly increased hypoxemic burden due to non-specific drifts was associated with CVD (Fig. 3). While the association between the different compositions of hypoxemic burden and the prevalence of cardiovascular disease has not been investigated previously, recent studies have shown that T90 was an independent predictor of all-cause death in patients with stable chronic heart failure [24] and was associated with an increased incidence of cardiovascular death and fatal stroke [23, 48].

On the contrary, Adderley et al. have shown that in a study with 14,117 patients with T2D, those patients who develop OSA (diagnosed by relevant diagnostic clinical code) were at increased risk of cardiovascular disease (adjusted hazard ratio 1.54, 95% CI [1.32, 1.79]) [49]. Also, Strausz et al. observed in a longitudinal population-based study of three cohorts with a cumulative 36,963 individuals that OSA (diagnosed by ICD codes) increased the risk for coronary heart disease independently of other risk factors in patients with T2D (hazard ratio 1.36, 95% CI [1.05 to 1.76]) [50]. In the present cross-sectional study, for T90 due to acute oxygen desaturation events there was no association with CVD. ODI and desaturation depth were also not associated with CVD in the adjusted models.

Our findings did not verify a previous analysis of a smaller DIACORE-SDB sub-study set, including 679 patients, where AHI was associated with CVD independently of other known risk factors [32]. The smaller cohort size may have suffered from sample bias. Also, in contrast to CKD, in the present analysis, the prevalence of CVD did not show a linear dose-response relationship. As the DIACORE patients exhibit many comorbidities that contribute to hypoxemia beyond SDB such as COPD and obesity [26], in the present, larger cohort other comorbidities and cardiovascular risk factors like age, obesity, high LDL levels, and smoking history predominate. In the present study, we have observed elevated odds for T90_desaturation_ in the univariable but not in the adjusted analyses.

### Strengths and limitations

Strengths of our study include the large sample size and the detailed phenotyping, providing comprehensive information about the participants’ lifestyle factors, and enabling adjustment for the main known risk factors for chronic kidney and cardiovascular disease.

The present study is subject to some limitations. First, our cross-sectional analysis cannot establish causal relationships between hypoxemic burden and CKD or CVD. Longitudinal data are required to establish causality and the potential predictive value of hypoxemic burden. Second, while we adjusted for many potential confounders, we cannot exclude the possibility of unmeasured confounders. Third, the study relied on physician-validated self-reported CVD diagnosis; thus, some cases of CVD may have been missed, and the potential association between nocturnal hypoxemic burden and CVD may have been underestimated. Fourth, the DIACORE study did not collect information about lung disease or chest X-rays; the non-specific hypoxemic burden observed in our study may partly reflect lung disease, pulmonary congestion, or ventilation-perfusion mismatches [26]. Lastly, our study was only conducted on patients with Caucasian ethnicity. Therefore, generalizability and extrapolation to other populations with T2D is limited.

## Conclusion

In summary, while hypoxemic burden due to oxygen desaturations and the magnitude of desaturation depth was significantly associated with CKD in a dose-response relationship, for CVD we observed a thresholdeffect and only severly and very severly increased hypoxemic burden due to non-specific drifts was associated with CVD. Thus, specific types of hypoxemic burden may differently be related to micro- and macrovascular disease. For CVD, concomitant risk factors and comorbidities, highly prevalent in patients with T2D and with SDB, seem to remain the predominant determinants for cardiovascular events and thus deserve intensive management. Yet, these findings emphasize the necessity to investigate whether there are causal mechanisms underlying the association between hypoxemic burden and CKD/CVD and how T2D influences this association. Further studies on the effect of CPAP therapy on microvascular disease are warranted. If longitudinal studies demonstrate causal relationships, the different compositions of nocturnal hypoxemic burden derived from overnight oximetry and quantified using a fully automated computer algorithm could be used as a simplified diagnostic and/or prognostic tool in patients with T2D for micro- and macrovascular diseases.

### Supplementary Information

Supplementary material 1. Table S1. Excerpt of the online questionnaire asked at the baseline visit and translated into English. Table S2: Baseline characteristics of the 1247 patients according to the severity of oxygen desaturation index and desaturation depth. Table S3. Univariate linear regression models for the association between hypoxemic burden parameters and serum creatinine, urine albumin-creatinine ratio and eGFR.

## Data Availability

No datasets were generated or analysed during the current study.
