# Supplementary material for: Nocturnal hypoxemic burden and micro- and macrovascular disease in patients with type 2 diabetes
Source: Cardiovasc Diabetol. 2024 Jun 6;23:195. doi: 10.1186/s12933-024-02289-w (PMC11157751; doi:10.1186/s12933-024-02289-w)
Supplement: Supplementary file 1 — Supplementary material 1. Table S1. Excerpt of the online questionnaire asked at the baseline visit and translated into English. Table S2: Baseline characteristics of the 1247 patients according to the severity of oxygen desaturation index and desaturation depth. Table S3. Univariate linear regression models for the association between hypoxemic burden parameters and serum creatinine, urine albumin-creatinine ratio and eGFR. [file 12933_2024_2289_MOESM1_ESM.docx]

## Online data supplement

**Online supplement table S1.** Excerpt of the online questionnaire asked at the baseline visit and translated into English.

| - Have you experienced pain or discomfort in your chest? |
| --- |
| - Have you suffered a heart attack? |
| - Did you get a heart catheterization? |
| - Did you get a heart surgery? If yes, what surgery was it? |
| - Did you get a contrast administration to depict the arteries of the legs? |
| - Did you get a surgery of your legs to improve blood flow? |
| - Did you have a stroke? Was it a cerebral bleeding? |

**Online supplement table S2:** Baseline characteristics of the 1247 patients according to the severity of oxygen desaturation index and desaturation depth.

| **Variables** | **ODI**  **< 15/h** | **ODI**  **≥ 15/h** | **P value** | **Desaturation depth**  **Q1-2**  **[0 – 2.7 %]** | **Desaturation depth**  **Q3-4**  **[> 2.7 %]** | **P value** |
| --- | --- | --- | --- | --- | --- | --- |
| n (%) | 937 (75.1) | 310 (24.9) |  | 616 (49.4) | 601 (48.2) |  |
| Age [years] | 66 ± 9 | 68 ± 8 | 0.005 | 66 ± 9 | 67 ± 8 | 0.160 |
| Sex (female), n (%) | 419 (44.7) | 95 (30.6) | <0.001 | 273 (44.3) | 228 (37.9) | 0.024 |
| BMI [kg/m^2^] | 30.4 ± 5.3 | 32.6 ± 5.3 | <0.001 | 30.1 ± 5.1 | 31.9 ± 5.5 | <0.001 |
| Waist-hip ratio | 0.94 ± 0.08 | 0.98 ± 0.08 | <0.001 | 0.94 ± 0.08 | 0.96 ± 0.08 | <0.001 |
| Former or current smokers, n (%) | 513 (54.7) | 191 (61.6) | 0.035 | 336 (54.5) | 344 (57.2) | 0.344 |
| High alcohol intake, n (%) | 252 (26.9) | 106 (34.2) | 0.014 | 161 (26.1) | 190 (31.6) | 0.035 |
| Physical inactivity, n (%) | 509 (54.3) | 192 (61.9) | 0.019 | 333 (54.1) | 353 (58.7) | 0.100 |
| HbA1c [mmol/mol] | 48 [43; 55] | 50 [44; 56] | 0.065 | 48 [43; 55] | 49 [44; 56] | 0.095 |
| T2D duration [years] | 7.5 [3.8; 13.7] | 9.6 [4.8; 15.4] | 0.004 | 7.7 [3.8; 13.4] | 8.7 [4.1; 14.9] | 0.035 |
| Systolic BP [mmHg] | 138 ± 18 | 141 ± 17 | 0.032 | 138 ± 17 | 140 ± 18 | 0.015 |
| Diastolic BP [mmHg] | 76 ± 10 | 76 ± 10 | 0.148 | 76 ± 10 | 76 ± 10 | 0.421 |
| Hypertension, n (%) | 387 (41.4) | 158 (51.0) | 0.003 | 256 (41.7) | 279 (46.4) | 0.097 |
| Serum creatinine [mg/dl] | 0.87 [0.74; 1.02] | 0.93 [0.80; 1.14] | <0.001 | 0.87 [0.75; 1.02] | 0.90 [0.76; 1.07] | 0.005 |
| eGFR-CDKepi [ml/min/1,73m²] | 84 [70; 94] | 78 [61; 91] | <0.001 | 84 [70; 94] | 81 [67; 92] | 0.015 |
| uACR [mg/g] | 8.8 [4.6; 24.5] | 9.9 [4.5; 35.9] | 0.153 | 8.5 [4.2; 23.3] | 10.0 [4.7; 30.0] | 0.028 |
| LDL [mg/dl] | 120 ± 36 | 116 ± 38 | 0.071 | 121 ± 37 | 117 ± 36 | 0.039 |
| HDL [mg/dl] | 54 ± 16 | 52 ± 15 | 0.020 | 55 ± 16 | 53 ± 15 | 0.048 |
| Mean SpO_2_ [%] | 93 ± 2 | 92 ± 2 | <0.001 | 93 ± 2 | 92 ± 2 | <0.001 |
| Min SpO_2_ [%] | 82 [80; 84] | 79 [74; 82] | <0.001 | 82 [81; 85] | 80 [75; 82] | <0.001 |
| Sleep efficiency [%] | 0.98 [0.88; 1.00] | 0.97 [0.89; 1.00] | 0.910 | 0.98 [0.88; 1.00] | 0.99 [0.88; 1.00] | 0.763 |
| T90 [min] | 8.2 [1.0; 39.0] | 45.0 [21.9; 123.0] | <0.001 | 4.5[0.6; 30.6] | 28.9 [11.1; 89.5] | <0.001 |
| T90_non-specific_ [min] | 2.1 [0.0; 28.2] | 9.5 [1.3; 51.1] | <0.001 | 1.5 [0.0; 20.7] | 6.5 [0.7; 49.0] | <0.001 |
| T90_desaturation_ [min] | 3.2 [0.6; 10.4] | 30.7 [16.6; 63.9] | <0.001 | 2.0 [0.4; 6.9] | 18.6 [7.4; 39.7] | <0.001 |
| ODI [events/h] | 4 [2; 8] | 23 [18; 33] | <0.001 | 4 [2; 7] | 13 [7; 22] | <0.001 |
| AHI [events/h] | 7 [4; 11] | 27 [21; 39] | <0.001 | 6 [4; 11] | 16 [9; 26] | <0.001 |
| Excessive daytime sleepiness, n (%) | 65 (7.0) | 24 (7.8) | 0.613 | 49 (8.0) | 39 (6.5) | 0.332 |

Results are provided as mean ± standard deviation for normally distributed and as median [interquartile range] for non-normally distributed variables.

AHI: apnoea-hypopnoea index, BMI: body-mass index, BP: blood pressure, eGFR-CDKepi: estimated glomerular filtration rate calculated using the CKD-EPI equation, HbA1c: hemoglobin A1c, HDL: high density lipoprotein, LDL: low density lipoprotein, ODI: oxygen-desaturation index, SpO_2_: arterial oxygen saturation; T90: night-time spent with oxygen saturation <90%, T90_desaturation_: T90 associated with acute oxygen desaturation events accompanied by resaturation, T90_non-specific_: T90 associated with non-specific and non-cyclic drifts in SpO_2_ or incomplete resaturation, uACR: urine albumin-creatinine ratio, Q: quartile.

High alcohol intake defined as ≥3/drinks per week; excessive daytime sleepiness defined as Epworth Sleepiness Scale ≥11; physical inactivity defined as light activity ≤2 times/week; hypertension defined as blood pressure ≥140/90 mmHg; sleep efficiency: sleeping time per time in bed.

**Online supplement table S3.** Univariate linear regression models for the association between hypoxemic burden parameters and serum creatinine, urine albumin-creatinine ratio and eGFR.

|  | **Serum creatinine** | | **Urine albumine-creatinine ratio** | | **eGFR** | |
| --- | --- | --- | --- | --- | --- | --- |
| **Variable** | **Regression coefficient B (95% CI)** | **P value** | **Regression coefficient B (95% CI)** | **P value** | **Regression coefficient B (95% CI)** | **P value** |
| T90 cont. | 0.001 (0.000; 0.001) | **<0.001** | 0.218 (0.034; 0.402) | **0.020** | -0.029 (-0.041; -0.016) | **<0.001** |
| T90 Q12 vs. Q34 | 0.069 (0.030; 0.108) | **<0.001** | 50.234 (19.159; 81.337) | **0.002** | -4.260 (-6.386; -2.133) | **<0.001** |
| T90non-specific cont. | 0.000 (0.000; 0.001) | **<0.001** | 0.229 (0.022; 0.437) | **0.031** | -0.027 (-0.041; -0.013) | **<0.001** |
| T90_non-specific_ Q12 vs. Q34 | 0.046 (0.008; 0.085) | **0.019** | 52.151 (21.068; 83.234) | **0.001** | -3.878 (-6.007; -1.750) | **<0.001** |
| T90_desaturation_ cont. | 0.002 (0.001; 0.002) | **<0.001** | 0.713 (0.182; 1.24) | **0.008** | -0.088 (-0.124; -0.053) | **<0.001** |
| T90_desaturation_ Q12 vs. Q34 | 0.069 (0.031; 0.108) | **<0.001** | 34.591 (3.430; 65.751) | **0.030** | -4.219 (-6.345; -2.092) | **<0.001** |
| Desaturation depth cont. | 0.044 (0.011; 0.076) | **0.008** | 12.72 (-13.81; 39.24) | 0.347 | -1.916 (-3.713; -0,120) | **0.037** |
| Desaturation depth Q12 vs. 34 | 0.054 (0.015; 0.093) | **0.006** | 16.02 (-15.68; 47.71) | 0.322 | -2.39 (-4.53; -0.244) | **0.029** |
| ODI cont. | 0.004 (0.003; 0.006) | **<0.001** | 0.800 (-0.501; 2.101) | 0.228 | -0.191 (-0.279; -0.103) | **<0.001** |
| ODI < vs. ≥ 15/h | 0.102 (0.058; 0.147) | **<0.001** | 8.571 (-27.678; 44.819) | 0.643 | -5.119 (-7.578; -2.660) | **<0.001** |
